# Kallikrein/K1, Kinins, and ACE/Kininase II in Homeostasis and in Disease Insight From Human and Experimental Genetic Studies, Therapeutic Implication

**DOI:** 10.3389/fmed.2019.00136

**Published:** 2019-06-27

**Authors:** Francois Alhenc-Gelas, Nadine Bouby, Jean-Pierre Girolami

**Affiliations:** ^1^INSERM U1138-CRC, Paris, France; ^2^CRC-INSERM U1138, Paris-Descartes University, Paris, France; ^3^CRC-INSERM U1138, Sorbonne University, Paris, France; ^4^I2MC-INSERM U1048, Toulouse, France

**Keywords:** angiotensin-converting enzyme, kallikrein (tissue), kinins, vasodilation, genetic human, genetic mouse models, Ischemic heart disease, diabetic nephropathy

## Abstract

Kallikrein-K1 is the main kinin-forming enzyme in organs in resting condition and in several pathological situations whereas angiotensin I-converting enzyme/kininase II (ACE) is the main kinin-inactivating enzyme in the circulation. Both ACE and K1 activity levels are genetic traits in man. Recent research based mainly on human genetic studies and study of genetically modified mice has documented the physiological role of K1 in the circulation, and also refined understanding of the role of ACE. Kallikrein-K1 is synthesized in arteries and involved in flow-induced vasodilatation. Endothelial ACE synthesis displays strong vessel and organ specificity modulating bioavailability of angiotensins and kinins locally. In pathological situations resulting from hemodynamic, ischemic, or metabolic insult to the cardiovascular system and the kidney K1 and kinins exert critical end-organ protective action and K1 deficiency results in severe worsening of the conditions, at least in the mouse. On the opposite, genetically high ACE level is associated with increased risk of developing ischemic and diabetic cardiac or renal diseases and worsened prognosis of these diseases. The association has been well-documented clinically while causality was established by ACE gene titration in mice. Studies suggest that reduced bioavailability of kinins is prominently involved in the detrimental effect of K1 deficiency or high ACE activity in diseases. Kinins are involved in the therapeutic effect of both ACE inhibitors and angiotensin II AT1 receptor blockers. Based on these findings, a new therapeutic hypothesis focused on selective pharmacological activation of kinin receptors has been launched. Proof of concept was obtained by using prototypic agonists in experimental ischemic and diabetic diseases in mice.

Kallikrein-K1 is the main kinin-forming enzyme in organs (including blood vessels) in resting condition whereas angiotensin I-converting enzyme/kininase II (ACE) is the main kinin-inactivating enzyme in the circulation (being present in both endothelium and plasma) (1–3). Both ACE and kallikrein have other, non-kinin related physiological actions, which are especially well-documented for ACE, first known as the angiotensin I-activating/angiotensin II-generating enzyme [(4), Figure 1].

**Figure 1:**
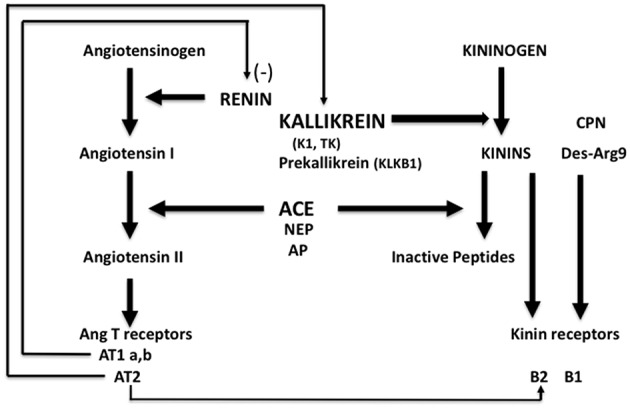
Schematic representation of the renin-angiotensin and kallikrein-kinin systems with physiological interrelation. ACE, angiotensin-converting enzyme/kininase II; NEP, neutral-endopeptidase; AP, aminopeptidase P; CPN, carboxypeptidase N.

Bioavailability of kinins in organs depends mainly on the balance between activities of K1 and ACE, locally. Bioavailability of angiotensin II depends on renin but also on ACE which is the only enzyme, in the human species, releasing angiotensin II from angiotensin I in the circulation. While early conception of regulation of the circulation was based on endocrine action of the renin-angiotensin system governed by renal renin secretion and autocrine/paracrine activity of the kallikrein-kinin system in organs where K1 is synthesized, subsequent research corrected, and expanded this concept. Indeed, ACE is not uniformly synthesized in the vasculature and its abundance displays strong vessel and organ specificity. Vascular ACE content is high in the lung and very low in the kidney and to a lesser extent the heart (5–7). Accordingly, bioavailability of angiotensin II is reduced in heart and kidney compared to other organs while action of locally produced kinins is potentiated in these organs. Both contribute to limiting vasoconstriction and maintaining high blood flow, especially in the kidney (5–8). This is the anatomical and physiological basis of the role of ACE in homeostasis, and of its role in disease (see below). The action of ACE adds a paracrine component to the renin-angiotensin system, especially active in the lung but downregulated in the heart and kidney.

Conversely, the kallikrein-kinin system has been shown to have an endocrine component based, in man, on renal (and perhaps also arterial) secretion of K1 (9). In rodents, circulating K1 comes mainly from salivary glands, which are well-developed in these animals (10). Endocrine action of K1 remains however poorly documented compared to local paracrine action of the enzyme in kidney, arteries, and exocrine glands.

Recent research, based mainly on human and animal genetic studies has documented the largely unsuspected physiological role of K1 in the circulation and has also refined understanding of the role of ACE/kininase II.

## The Physiological Role of K1

Kallikrein-K1 belongs to a large family of genetically and structurally related proteases but is the sole enzyme in the family able to catalyze the hydrolysis of kininogens and release kinins (10, 11). In absence of specific inhibitors of K1 suitable for *in vivo* studies the issue of the physiological role of the enzyme was addressed through inactivation of the *Klk1* gene in the mouse and discovery of a loss of function polymorphism of K1 in man (12).

**Figure 2:**
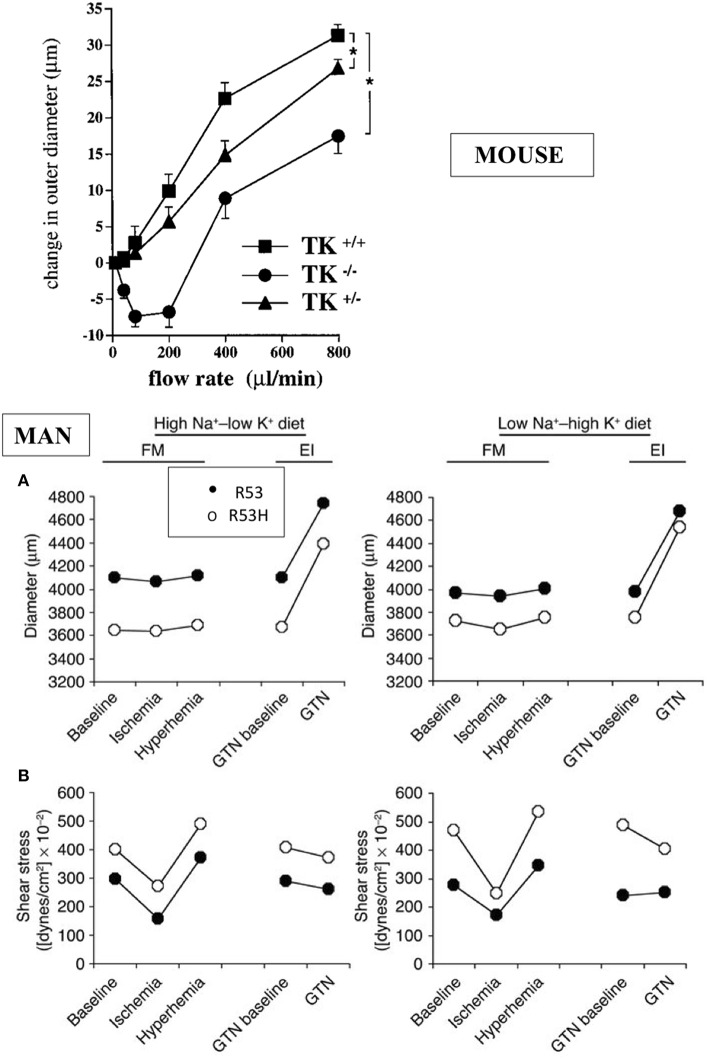
Arterial dysfunction in K1 deficient mice and human subjects partially deficient in K1 activity. Upper graph: impairment of flow-induced vasodilatation in carotid artery of K1 deficient mice. Homozygote (TK^−/−^) and heterozygote (TK^+/−^) mice with inactivated K1 gene compared to littermate wild type animals (TK^+/+^). ^*^p < 0.05 compared to littermate TK^+/+^. Note paradoxical vasoconstrictor response at low flow rate in TK^−/−^ and partial defective phenotype of TK^+/−^ mice (see text for discussion). Reproduced from Bergaya et al. (15). Lower panels: arterial dysfunction in subjects carrying the defective R53H mutation of K1 (heterozygote) and having roughly 50% K1 activity level of non-mutated, homozygous R53 subjects. Subjects were studied by vascular echotracking of brachial artery in basal condition, during hand ischemia and reactive hyperhemia and after nitroglycerin (GTN) administration. Study was repeated at contrasted dietary Na/K intake. Low Na-High K stimulates K1 synthesis in the kidney. Note increase in sheer stress **(A)** in R53H subjects with paradoxical reduction of arterial diameter **(B)**. Reproduced from Azizi et al. (16). Observations in both man and mouse are indicative of endothelial dysfunction (see text).

The K1 deficient mice grow and reproduce normally. They have normal blood pressure in resting condition but display arterial functional abnormalities (13–15). The mice have severe impairment of flow dependent vasodilatation, a prominent feature of arterial physiology ensuring proper delivery of blood to organs during variation in cardiac output [(13, 15), Figure 2]. Flow dependent dilatation is partly a kinin and kinin-B2 receptor mediated process with kinins released locally by arterial K1 from circulating and endothelial-bound kininogen acting in a paracrine/autocrine manner (15). This occurs in both buffer and resistance arteries (15, 17). The vasodilator effect of the angiotensin II AT2 receptor is also impaired in K1 deficient mice, extending to arteries observation of functional coupling between AT2 and B2 receptors originally made in the kidney and establishing role of K1 and kinins in this coupling (18–20). Overall, arterial functional abnormalities in K1 deficient mice are consistent with endothelial dysfunction, secondary to kinin deficiency (15, 17, 18).

Interestingly, heterozygote deficient mice with roughly 50% K1 activity also display a defective arterial phenotype, indicating that K1 activity level in arteries (where K1 is synthesized in low abundance compared to several other organs) is a critical factor for arterial function (15). This has been further documented in man (see below).

Despite having functional arterial abnormalities, K1 deficient mice have normal blood pressure regulation and conserved circadian variation in resting condition (13, 21). While K1 was initially discovered as a hypotensive agent, the enzyme, at physiological level, either does not significantly influences vascular resistance or its action is offset by compensatory regulations. However, K1 has anti-hypertensive action in at least one pathological setting. The K1 deficient mice, when challenged with salt and aldosterone, a treatment increasing K1 synthesis in wild-type mice, develop hypertension (see below).

While K1 is synthesized in high abundance in epithelial cells in the kidney (in the distal tubule) and in other exocrine glands, K1 deficient mice display only minor renal epithelial abnormalities related to calcium and potassium handling, with mild hypercalciuria and delayed response to dietary potassium load (13, 22, 23).

Interestingly, it has been possible to extent to man the observations made in K1 deficient mice. Indeed K1 activity level is genetically determined in man and this is caused, at least in part, by a loss of function polymorphism of the enzyme substituting an histidine for an arginine in a substrate binding subsite of the active site (R53H) (24–26). The mutation has an allele frequency of 0.03 and only heterozygote subjects (7% of white population) could be studied (26). But these partially deficient K1 subjects display functional arterial abnormalities evidenced by vascular echotracking study. These abnormalities, increase in sheer stress with paradoxical inward arterial remodeling, are, like in K1 deficient mice, suggestive of arterial endothelial dysfunction [(16), Figure 2]. The R53H subjects also display minor epithelial abnormalities related to calcium or potassium handling, similar to the mouse (27, 28).

Mouse and human genetic studies thus allowed recognizing, consistently in both species, the physiological role of K1 in the circulation and its role in the kidney.

Overall, in resting condition, K1 deficiency induces only minor vascular and renal abnormalities in young individuals, albeit it is not known whether these defects can eventually translate into altered life expectancy. But, in pathological situations resulting from hemodynamic, ischemic, or metabolic insult to the cardiovascular system and the kidney K1 exerts critical end-organ protective action and K1 deficiency results in severe worsening of the conditions, at least in the mouse. This is being discussed below.

## Physiological Role of ACE/Kininase II in the Circulation and in the Kidney

The ACE is an endothelial ectoenzyme, also secreted as a so-called soluble form in plasma by enzymatic cleavage separating the core molecule from its C-terminal transmembrane anchor (5, 29). Conversion of angiotensin I and inactivation of kinins are believed to occur mainly on the endothelial surface albeit, as said above, there is strong heterogeneity in endothelial ACE content along the vascular tree, with physiological consequence.

**Figure 3:**
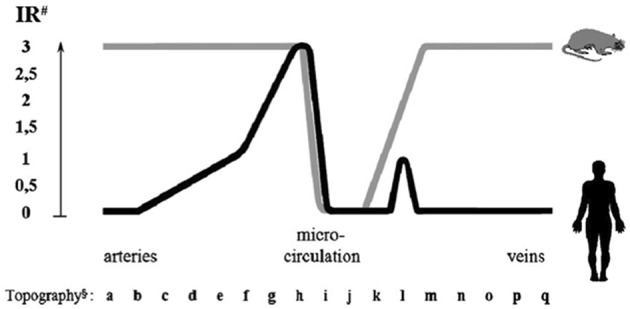
Endothelial ACE distribution along the human and rat vascular tree. Endothelial ACE is heterogeneously distributed in humans (black line). Large arteries contain no or little ACE while maximal ACE content is observed in small muscular arteries and arterioles. Only a fraction of capillaries network contains ACE. Venules and veins contain no or little ACE except some muscular veins in upper and lower extremities. Exceptions to this general scheme are the renal circulation and the pulmonary capillary network (not shown, see text for discussion). In the rat (gray line) ACE is more homogeneously distributed in arteries and veins, while only a fraction of capillaries contains ACE, like in man. ACE is also dowregulated in renal arterioles (not shown). ^#^Immunoreactivity (IR) shown is scored from 0 (no reactivity detectable) to 3 (maximal reactivity) and refers to defined mAb concentrations. ^§^Localization of endothelial cells: (a) aorta ascendens, arcus aortae, aorta descendens/thoracalis/abdominalis; (b) a. brachiocephalica, a. pulmonalis, a. iliaca communis, a. carotis communis; (c) a. iliaca interna/externa, a. carotis interna/externa, a. subclavia; (d) truncus coeliacus, a. renalis, a. mesenterica sup./inf., a. axillaris, a. femoralis; (e) a. brachialis, a. poplitea, a. tibialis ant./posterior, a. ulnaris, a. radialis, a. peronea; (f) a. dorsalis pedis, a. digitalis plantaris/palmaris; (g) small peripheral arteries; (h) pre-arterioles, arterioles; (i) capillaries; (j) venules; (k) small peripheral veins; (l) v. digitalis plantaris, v. dorsalis pedis, v. saphena parva, v. saphena magna; (m) v. digitalis palmaris, v. tibialis, v. poplitea; (n) v. axillaris, v. femoralis, v. jugularis ext./int; (o) v. iliaca int./ext., v. subclavia; (p) v. iliaca communis, v. brachiocephalica, v. renalis; (q) v. cava sup./inf. Reproduced from Metzger et al. (7).

In man, ACE is mainly synthesized in small muscular arteries and in arterioles. Immunoreactive ACE is low or undetectable in large arteries and in veins. Only a fraction of capillaries in organs contains ACE, with two divergent exceptions, the lung and the kidney [(7), Figure 3].

In the pulmonary capillary bed high and homogeneous ACE content ensures conversion of angiotensin I and inactivation of bradykinin, both produced in the renal circulation and post-renal venous system and conveyed to the lung (7). In the renal circulation on the opposite, low or undetectable endothelial ACE content in face of high renin and angiotensin I concentrations limits local angiotensin II formation and consequently renal vasoconstriction (6, 7, 30). This is consistent with early studies documenting reduced angiotensin I conversion in the renal circulation (8). Kinins are also formed in abundance in the kidney interstitium from K1 synthesized in the distal tubule and released in both blood and urine. Lack of ACE in renal vessels potentiates the vascular action of kinin. Plasma ACE contributes to some extent to angiotensin I conversion and kinin inactivation in the renal circulation but cannot fully substitute for endothelial ACE in these functions (30, 31).

Bradykinin is the best substrate for ACE, i.e., the substrate with the most favorable kinetic parameters. Somatic ACE, which results from duplication of an ancestral gene, has two active sites and bradykinin is the best substrate for both ACE active sites (5, 32–36). ACE is not the only enzyme able of inactivating kinins in the circulation but because of its kinetic parameters it plays a major role, physiologically. Accordingly, kinins produced in organ interstitium by K1 in physiological or pathological conditions or in plasma by the other kallikrein, (pre)kallikrein (KLK1) when activated in pathological situations are quickly inactivated locally, except in the kidney (37, 38).

Interestingly, both theoretical and experimental evidences suggest that *in vivo* variations in ACE level influence largely bradykinin concentration but have little effect on angiotensin II concentration (39–41). This is because of a superimposed negative regulatory loop in the renin-angiotensin system between renal angiotensin II concentration and renin secretion, offsetting any ACE-related increase in angiotensin II, at least in the kidney.

Angiotensin I-converting enzyme has other substrates, at least *in vitr*o, for which the enzyme displays less favorable kinetic parameters than bradykinin or angiotensin II, like substance P and LHRH (35). It is not clear whether substance P and LHRH are metabolized by ACE physiologically (42). But hydrolysis of the hematopoietic peptide N-AcSDKP by the N-terminal active site of ACE has been documented both *in vitro* and *in vivo* (43). This peptide has been reported to have cardiac and renal anti-fibrotic action in experimental models (44).

Angiotensin I-converting enzyme, especially its N-terminal active site, can hydrolyse *in vitro* the Alzheimer amyloid Aβ-peptide that is believed to be causally involved in Alzheimer disease (45, 46). But, while some evidence for a role of ACE in limiting progression of experimental neurodegenerative diseases has been obtained in animals the role of ACE in Alzheimer disease remains controversial, despite being suggested by some clinical studies (45–48).

Angiotensin I-converting enzyme has also been shown to behave as a signaling molecule in cells, independently of its enzymatic activity, through phosphorylation of a serine in the short intracellular domain. Signaling is triggered by ACE inhibitor binding, likely as a result of ACE conformational change and dimerization, involves CK2 kinase, mitogen-activated protein kinase kinase 7, and c-Jun N-terminal kinase and induces expression of some endothelial genes, including the *ACE* gene (49, 50). However, no physiological agonist of this pathway has been identified and its physiological role remains undocumented. The ACE signaling is however probably involved in the well-documented phenomenon of counter-regulatory increase in ACE synthesis during ACE inhibitor treatment (51). It has also been reported that ACE is involved in angiotensin II signaling *in vitro* in cultured cells but this observation remains of unknown physiological relevance (52).

Angiotensin I-converting enzyme levels are genetically determined in man. The above anatomical and physiological considerations are relevant to mechanistic issues pertaining to the well-documented role of this genetic variation in cardiovascular and renal diseases (see below).

## Role of K1, Kinins, and ACE/Kininase II in Cardiovascular and Renal Diseases

Through pharmacological or genetic inactivation of K1 and kinin receptors in animals, manipulation of ACE/kininase II gene expression or activity as well as clinical studies it was shown that kinin release during hemodynamic, metabolic, or ischemic insult reduces organ damage, especially in the heart and kidney. This has been reviewed previously (37, 53, 54).

### Experimental Studies, Cardiac and Peripheral Ischemia, Diabetes, Hypertension

**Figure 4:**
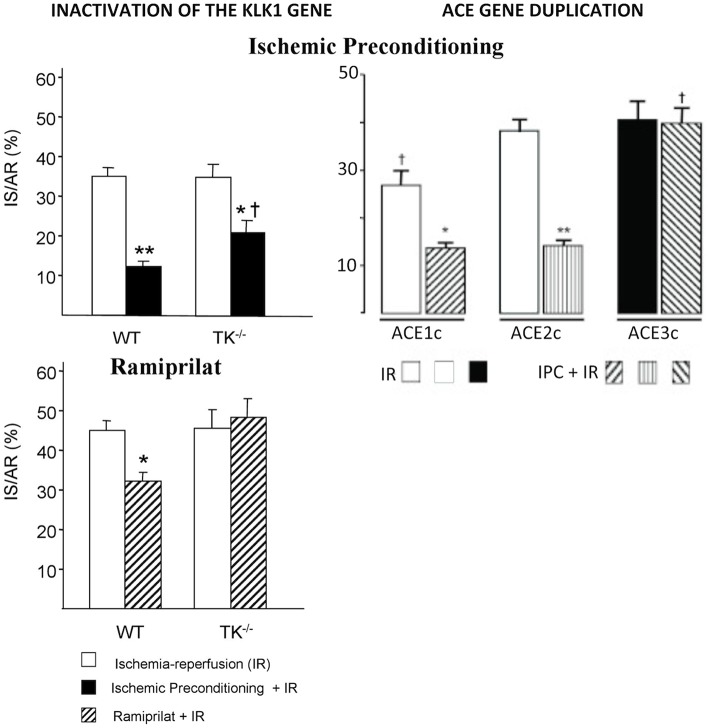
Loss of the cardioprotective effect of ischemic pre-conditioning in mice with an inactivated *Klk1* gene or mice carrying a duplication of the *ACE* gene in cardiac ischemia-reperfusion. IS/AR: infarct size relative to area at risk. IR, ischemia-reperfusion; IPC, ischemic pre-conditioning followed by IR; WT, wild type; TK^−/−^, K1 deficient mice; ACE1c, mice with a single ACE gene copy; ACE2c, mice with two ACE gene copies (wild type); ACE3c, mice with three ACE gene copies having roughly 140% ACE activity level of wild-type mice. Note also loss of cardioprotective effect of the ACE inhibitor Ramiprilat in K1 deficient mice. ^*^p < 0.05, ^**^p < 0.01 compared to IR; ^†^*p* < 0.05 compared to littermate WT or ACE2c (WT). Adapted from Griol-Charhbili et al. (55) and Messadi et al. (56).

In cardiac ischemia-reperfusion in mice, genetic deficiency in K1 suppresses cardioprotective mechanisms limiting necrosis, like ischemic preconditioning [(55), Figure 4]. Moreover, deficiency in K1 abolishes the infarct-size reducing effect of an ACE inhibitor or an angiotensin II AT1 blocker and also the effect of the mitochondrial pore opening inhibitory drug cyclosporin A (55, 57, 58). Loss of the cardioprotective effect of ACE inhibitors in cardiac ischemia in K1 and kinin deficient mice is explained by the well-documented role of kinins in the beneficial effect of the drugs in this experimental model (59). Loss of the cardioprotective effect of AT1 blockers is also explained by kinin deficiency, because AT1 receptor inhibition increases renin secretion and angiotensin II production resulting in activation of the angiotensin II AT2 receptor. As discussed above, the AT 2 receptor is functionally coupled to K1 and kinins. This physiological pathway, inactivated in K1 or B2 receptor deficient mice, is shown here to play a prominent role in the effect of AT1 blockers in experimental cardiac ischemia (57, 60). For cyclosporin A, the mechanism of the permissive effect of K1 is less understood but may be related to direct mitochondrial action of the enzyme (58).

The effect of *Klk1* gene inactivation in acute cardiac ischemia is mimicked by duplication of the ACE gene, suggesting that kinin depletion plays a prominent role in organ damage in both genetic mouse models [(56, 61), Figure 4]. However, the deleterious cardiac effects of ACE gene duplication can be partially prevented by renin inhibition, indicating that both kinin depletion and enhanced local angiotensin II formation are involved in ACE-mediated cardiac damage in ischemia (56).

Cardiac or renal protective effect of kinins in ischemia has also been documented by studies in kininogen or kinin receptor deficient animals and in animals treated with kinin receptor antagonists (62). Kinins are potent endothelial activators promoting vasodilatation of collateral arteries thus increasing distal blood flow delivery and limiting thrombus extension by releasing endothelial mediators inhibiting platelet aggregation and triggering fibrinolysis (15, 63–66). Moreover, K1 and kinins exert cytoplasmic and mitochondrial actions decreasing oxidative stress in tissues (58, 67).

In chronic ischemic and post-ischemic diseases, vascular and cellular actions of kinins also prevent end-organ damage. In the ischemic hindlimb of diabetic mice or rats submitted to femoral ligation, an experimental situation where post-ischemic angiogenesis is impaired by diabetes, K1 and kinins exert vasodilatory and proangiogenic effects restoring distal blood flow. Deficiency in K1, kinins, or kinin receptors worsens hindlimb ischemia (68–70). Kinins exert proangiogenic effect through mobilization of progenitor cells with endothelial potentiality and also through mobilization and activation of macrophages (a so-called inflammatory response) (71–73).

In post-ischemic heart failure secondary to irreversible coronary occlusion, kinins prevent excess ventricular remodeling, which has deleterious electrophysiological and hemodynamic consequences. Deficiency in K1 or kininogen exaggerates remodeling and decreases long-term survival (74, 75). K1 is synthesized in the heart, although it is not clear whether synthesis occurs only in coronary vessels or also in cardiomyocytes. Kinins are involved in the cardioprotective effect of ACE inhibitors in post-ischemic heart failure and also in the effect of the renin inhibitor Aliskiren (74, 76). For Aliskiren, the mechanism proposed involves drug-induced increase in cardiac K1 synthesis, kinin release, and kinin B2 receptor activation, independently of renin inhibition (76).

### Diabetes

**Figure 5:**
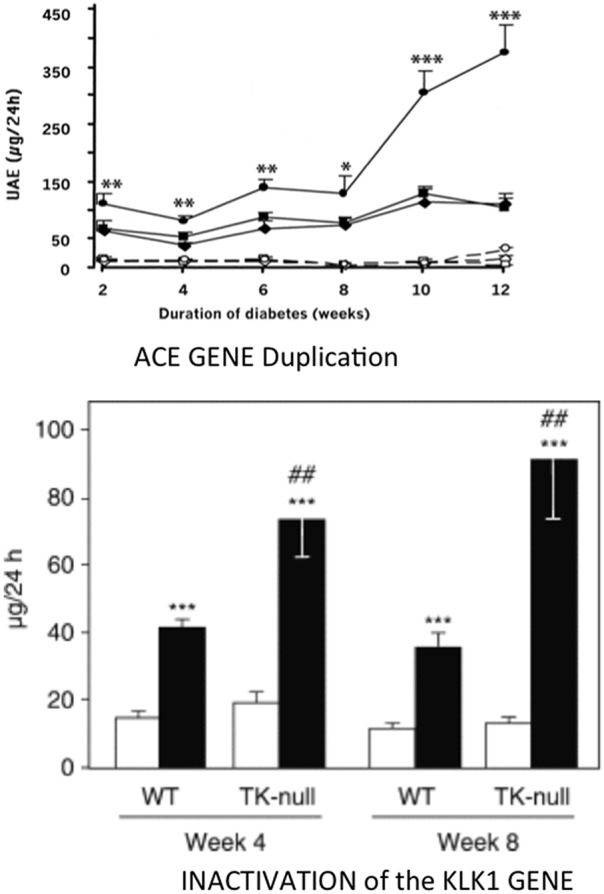
Aggravating effect of inactivating the *KLK1* gene or duplicating the *ACE* gene on development of diabetic nephropathy in diabetic mice. Ordinate in both graphs: urinary albumin excretion. Upper graph, ACE gene titration; open symbols, non-diabetic mice; filled symbols, diabetic mice. Diamonds, one-copy mice; squares, two-copy mice (wild-type); circles, three-copy mice. ^*^p < 0.05, ^**^p < 0.01, ^***^p < 0.001 compared to diabetic two-copy (WT) or one-copy mice. Lower graph: K1 gene inactivation. open bars control, non-diabetic mice; filled bars diabetic mice. ^***^p < 0.001 compared to non-diabetic mice. ^*##*^*p* < 0.01 compared to corresponding WT mice. Adapted from Bodin et al. (77) and Huang et al. (78).

Diabetes is a disease where the role of K1 and kinins in limiting organ damage has been especially well-documented, experimentally. In mice rendered diabetic, the synthesis of K1, kininogen, and kinin receptors increases rapidly in the kidney after the onset of hyperglycemia. Inactivation of the *Klk1* gene aggravates diabetic nephropathy [(77), Figure 5]. The same aggravating effect is observed by inactivating the kinin B2 receptor gene (79). Diabetic nephropathy is both a vascular and a renal disease secondary to chronic hyperglycemia and in humans it is strongly linked, already at its early stage, to enhanced risk of coronary heart disease. Diabetic nephropathy can also evolve into renal insufficiency, thus carrying dual cardiac and renal risk. Interestingly, in the Akita mouse with early onset insulinopenic diabetes, deficiency in kinin B2 receptor induces not only renal damage but also a generalized, pro-senescent phenotype of organs (80).

Synthesis and secretion of endothelial ACE increases in diabetes. Duplication of the ACE gene in insulinopenic mice accelerates development of diabetic nephropathy [(78), Figure 5]. As discussed above for cardiac ischemia, induction of similar, deleterious renal phenotypes in diabetes by either deficiency in K1 or overexpression of the *ACE* gene is consistent with kinin bioavailability having a major role in renal protection against hyperglycemia (77, 78). However, genetic increase in ACE can also potentiate angiotensin II formation locally and, for less understood reasons, angiotensin II action (56, 81). Angiotensin II is involved together with kinin depletion in the effect of ACE gene duplication, at least in the heart (56).

### Hypertension

An antihypertensive role of K1 has been repeatedly suggested after K1 was discovered by its hypotensive property (82). This role seems to be mainly exerted in the setting of salt and aldosterone excess, where renal K1 synthesis is stimulated, and may not be, or entirely be, kinin-mediated (83). K1, like other tubular proteases, may regulate sodium transporter activity in the kidney (53, 83). Deficiency in K1 has no effect in renovascular hypertension (one clip, one kidney), a renin rather than volume (salt) dependent type of hypertension (21).

Taken together, mice studies document that the physiological actions of K1 have, overall, beneficial consequence for preservation of end-organ trophicity and function in the settings of acute or chronic ischemia, chronic hyperglycemia and mineralocorticoid, and salt excess. The role of K1 is kinin-mediated in ischemia and in diabetes. ACE activity has, on the opposite, deleterious end-organ effect in ischemia or diabetes, through both local angiotensin II formation and kinin depletion.

### Clinical Studies, Genetic Variation in ACE/Kininase II Level, and Risk of Cardiovascular and Renal Diseases

Both ACE and K1 activity level are well-established quantitative genetic traits in man. This was first documented in familial transmission studies (24, 25, 84, 85). Genomic markers for these traits have been then identified. For K1, a frequent mutation in the active site (R53H, discussed above) is causally involved in the genetic variability of K1 activity (26). For ACE, an intronic insertion/deletion polymorphism is strongly associated with plasma and tissue ACE levels, although it is not clear whether this genomic variation is causally involved in the phenotype (through modulating mRNA stability and splicing) or is only a neutral marker in linkage disequilibrium with another, causal mutation (86–88). Despite extensive investigation of the ACE gene the putative causal mutation has however not been identified (5, 89).

Ìn any case, the genetic variation in ACE level has been, quickly after its discovery, associated with susceptibility to and prognosis of cardiovascular and renal diseases. First observation was made for myocardial infarction in a landmark European multicenter study where the subjects homozygote for the ACE D allele, the allele which is associated with higher ACE levels, were found to be at increased risk (90, 91). The association has been reproduced in several other studies but not in all adequately powered studies, although it was consistently observed in the setting of diabetes (5, 92). ACE is probably a weak genetic risk factor for myocardial infarction in the general population, but its effect is potentiated by diabetes and reciprocally. Interestingly, mice carrying a duplication of the ACE gene and having a modest genetic increase in ACE level similar to that observed in humans homozygote for the D allele display reduced myocardial tolerance to cardiac ischemia, thus, documenting causality and mechanism behind the proposed clinical association (56) (Figure 4).

**Figure 6:**
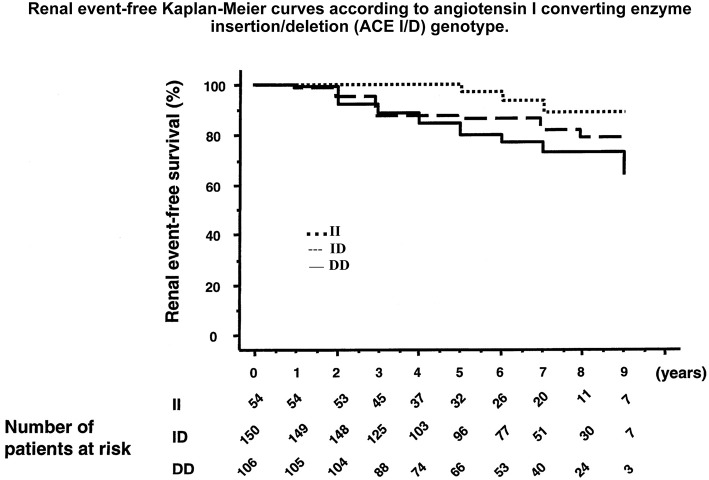
Association of the ACE gene ID polymorphism with development of diabetic nephropathy in patients with type 1 diabetes. Graph shows cumulative incidence of renal events (progression from physiological to pathological microalbuminuria or from a given stage of nephropathy to a higher stage) according to ACE genotype. D allele is co-dominantly associated with higher ACE levels. Reproduced from Hadjadj et al. (97). This study documents the role of the genetic variation in ACE level in susceptibility to and progression of diabetic nephropathy. Same observation was made in other patient populations, before and after this study. See text and Marre et al. (93) through Hadjadj et al. (100).

But, genetic variation in ACE level has been well-established as a risk factor for diabetic nephropathy, in type 1 diabetes. The association, originally found independently in the GENEDIAB study in Europe and at the Joslin clinic in the US, has been confirmed in the major cohort studies of type 1 diabetic patients, including the landmark DCTT/EDIC study [(92–100), Figure 6]. Hyperglycemic diabetic patients with genetically high ACE levels are at increased risk of developing nephropathy and the disease evolves more severely in these patients (92–100). Diabetic nephropathy is both a vascular and a renal disease and affected patients display, even at early stage of nephropathy clinically marked by elevated urinary albumin excretion and conserved renal function, increased incidence of cardiovascular events, especially myocardial infarction (101). Diabetic nephropathy was known to have a strong genetic determinism in type 1 diabetes (102). The ACE gene is the first recognized and so far best documented gene involved in the disease. Causality between genetically determined ACE level and renal involvement in diabetes was established by ACE gene titration (1 to 3 copies) in the mouse, as discussed above. Nephropathy develops faster in mice having three ACE gene copies and a moderate increase in ACE level, when the mice are rendered diabetic (78) (Figure 5).

In summary, through combined human genetic association studies and causality study in genetically modified mice ACE was established as a risk/prognosis factor for diabetic nephropathy in insulinopenic diabetes. As discussed above, both theoretical and experimental evidences suggest that reduced bioavailability of kinins is involved in the deleterious effect of ACE in diabetes. This is also supported by clinical physiology studies (103, 104).

On the other hand the defective K1 mutation was not found to be associated with cardiovascular and renal diseases so far, including in studies where ACE was associated. It can however be noted that because of the low allele frequency of the mutation only heterozygote subjects with partial deficiency in K1 activity were included in these studies.

## Therapeutic Development, Selective Agonism of Kallikrein- Kinin

Based on the organ-protective action of K1 and kinins in ischemia and diabetes, a new therapeutic approach to cardiovascular and renal diseases has been proposed, selective agonism of the kallikrein-kinin system. The topic has been reviewed previously (37, 105).

Rationale for developing pharmacological agonists of kallikrein-kinin is further based on the well-documented role of kinins in the therapeutic effect of both ACE inhibitors and angiotensin II AT1 receptor blockers, as discussed above. But, as K1 activity level is low in the cardiovascular system, kinins are only produced at slow rate in the circulation and ACE inhibitors, or angiotensin II AT1 blockers can only potentiate endogenously formed kinins. Low K1 activity and slow kinin formation might limit the therapeutic efficacy of current kinin-potentiating drugs (15, 37, 105).

Kallikrein cannot be easily targeted for pharmacological activation [albeit Aliskiren, a clinically approved compound originally designed as a renin inhibitor has been reported to stimulate K1 synthesis in the failing rat heart as discussed above, Koid et al. (76)]. Kallikrein gene therapy and additive transgenesis with the human *klk1* gene have been attempted in animals, with report of beneficial effects in cardiac or peripheral ischemic and diabetic diseases (68, 106–110). However, kallikrein gene therapy is difficult evaluating experimentally, in part because of species specificity in K1 activity and it is unlikely to be ever developed in clinical practice (105, 111). Bradykinin and other natural kinins, such as des- Arg^9^-bradykinin, the endogenous B1 receptor agonist, cannot be used pharmacologically as therapeutic agents as they are readily destroyed by peptidases, especially ACE or carboxypeptidase N, in the circulation (3).

Pseudo-peptide analogs of kinins have been synthesized. These compounds are potent kinin receptor agonists, selective for either the B1 or the B2 receptor and are resistant, by design, to peptidases action (112, 113). The agonists can be administered intravenously or chronically through osmotic micropumps (73, 114). The B2 agonist dose-dependently decreases blood pressure during acute administration but has no sustained hypotensive effect in chronic administration, probably as a consequence of counter-regulations. The B1 agonist has no effect on blood pressure (73, 114).

These prototypic agonists allowed establishing proof of concept for therapeutic efficacy of pharmacological kinin receptor activation in animals. They have also helped documenting the functions of the B1 and the B2 receptor in diseases because of their selectivity. Indeed, while the B2 receptor (B2R) is constitutively synthesized in the cardiovascular system and the kidney the B1 receptor (B1R) is considered as being only synthesized in pathological situations (115). Situations where B1R synthesis is induced include ischemia, chronic hyperglycemia, and also, interestingly, ACE inhibitor therapy (77, 115–118). Use of selective agonists in experimental diseases has further documented the physiological balance between the two receptor-subtypes and shown that the balance is strongly influenced by diabetes, especially in the heart.

**Figure 7:**
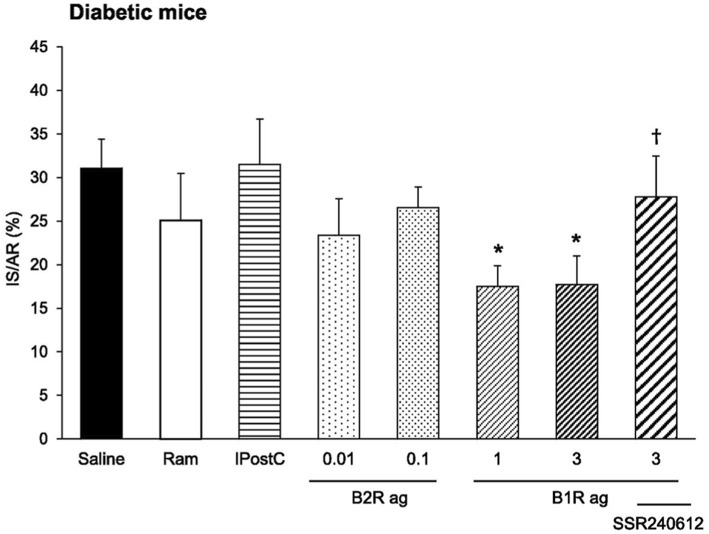
Cardioprotective effect of a pharmacological kinin B1 receptor agonist in cardiac ischemia-reperfusion in diabetic mice. IS/AR, infarct size relative to area at risk; B1R ag, kinin B1 receptor agonist; B2R ag, kinin B2 receptor agonist; Ram, ramiprilat; IpostC, ischemic post-conditioning; SSR240612, kinin B1 receptor antagonist. Note resistance of the diabetic heart to cardioprotective treatments otherwise efficient in non-diabetic mice (Ram, B2R ag and IpostC). Reproduced from Potier et al. (114).

The kinin receptor agonists have been tested in experimental ischemic and/or diabetic diseases in mice. Cardiac ischemia-reperfusion is an experimental model for human ischemic heart disease. A B2R agonist administered at reperfusion largely reduces infarct size (by 47%). A B1R agonist has no effect. However, if the mice are rendered diabetic prior to inducing ischemia the B2R agonist loses its cardioprotective effect. But in these diabetic mice, the B1R agonist becomes efficient and largely reduces infarct size (by 44%). Cardioprotective signalization involves, for both receptors, phosphoinositide three kinase/Akt and extracellular signal-regulated kinase 1/2, GSK- 3b inactivation and inhibition of mitochondrial pore opening [(114), Figure 7]. These observations suggest that B2R signaling is suppressed in the diabetic heart with compensatory induction of B1R, taking over cardioprotective signaling. Infarct size reducing effect of the B2R agonist is consistent with the role of the B2R in cardiac ischemia previously documented by using loss of function approaches. Interestingly, in this study, the B1R agonist was the only pharmacological agent tested displaying therapeutic effect in diabetic animals, as an ACE inhibitor, like the B2R agonist had no effect on infarct size (114) (Figure 7). Ischemic post-conditioning was also ineffective in diabetic animals. The mouse or rat diabetic heart is known to be resistant to established cardioprotective treatments. Interestingly, pharmacological B1R agonism is able to overcome this resistance (114).

Pharmacological kinin receptor agonism also displays therapeutic efficacy in chronic hindlimb ischemia in diabetic mice. Mechanisms involved here pertain to angiogenesis rather than tissue tolerance to ischemia. While, in non-diabetic mice femoral artery ligation does not induce sustained hindlimb ischemia and distal perfusion is recovered in a few days through neovascularization, in diabetic mice with defective neovascularization capacity the hindlimb remains ischemic. Treatment of diabetic mice with either a B1R or a B2R agonist for 2 weeks following artery ligation enhances neovascularization and restores distal blood flow. Monocyte/macrophage mobilization, VEGF synthesis, and mobilization of progenitor cells are involved in the angiogenic effect of the agonists post-ischemia (73).

But B1R or B2R activation in diseases may not always be beneficial. Indeed, in cerebral ischemia-reperfusion in mice B2R activation increases mortality, perhaps through peripheral hemodynamic effects. The B1R agonist has no effect in non-diabetic mice. But interestingly, in diabetic mice, B1R agonist given at reperfusion decreases brain infarct size and reduces neurological deficits, further documenting efficiency of B1R agonism in improving tolerance to ischemia of diabetic organs (116). In a study of skin wound healing, which can be an important clinical issue in diabetic patients, pharmacological B2R activation delayed healing in non-diabetic or diabetic mice, while B2R inhibition corrected the healing defect observed in diabetic mice. Activation of B1R had no effect on wound healing in either non-diabetic or diabetic mice, despite induction of B1R synthesis in the wounded diabetic skin (117).

Overall, mice studies document beneficial effects of B1 receptor agonism in ischemia in the setting of diabetes, consistently in the heart, brain, or hindlimb. B2R agonism has cardioprotective effect in cardiac ischemia and proangiogenic action in peripheral ischemia but deleterious effects in brain ischemia. Studies in diabetic nephropathy are still awaited. For retinopathy, role of kinins is controversial. Studies have suggested that some retinal vascular effects of B2R may be deleterious (119, 120). The issue might be clarified by gain of function studies with selective pharmacological agonists (37).

Results of experimental therapeutic studies should always be translated with caution to clinical situations, regarding both efficiency and tolerance of compounds tested. The present studies provide at least proof of concept for therapeutic action of selective pharmacological kinin receptor agonism. Kinin receptor agonists might have enhanced therapeutic efficacy compared to ACE inhibitors, especially in the diabetic heart. No unwanted effects, like hypotension, oedema, or abnormal psychomotor behavior suggestive of excessive pain suffering were observed during up to 2 weeks agonist treatment in the mice (73, 117). However, in the clinical setting, pharmacological B2R agonism might have serious unwanted side effects, including angioedema, pain or tumor development (37, 105, 121–123). This remains speculative. On the other hand, B1R agonism was consistently efficient in diabetic animals and had no detrimental or off-target effects in the experimental diseases studied so far. The B1R may not be involved in angioedema, contrary to the B2R. Indeed, selective blockade of the B2R, presumably resulting in enhanced kinin-induced B1R activation, is considered to improve outcome of angioedema crises. Clinical development of kinin receptor agonists, especially B1R, is worth considering.

## Author Contributions

All authors listed have made a substantial, direct and intellectual contribution to the work, and approved it for publication.

### Conflict of Interest Statement

The authors declare that the research was conducted in the absence of any commercial or financial relationships that could be construed as a potential conflict of interest.
